# Investigation of vehicles as potential pathways for between-farm transmission of influenza A virus in US dairy herds

**DOI:** 10.3168/jdsc.2025-0999

**Published:** 2026-03-19

**Authors:** N. Urie, N. Amey, E. Marshall, K. Abernathy, N. Wineland, J. Lombard

**Affiliations:** 1 Department of Clinical Sciences, College of Veterinary Medicine and Biomedical Sciences, Colorado State University, Fort Collins, CO 80523; 2 AgNext, College of Agricultural Sciences, Colorado State University, Fort Collins, CO 80523; 3 United States Department of Agriculture, Animal and Plant Health Inspection Service, Lansing, MI 48933; 4 Antimicrobial Use and Stewardship, California Department of Food and Agriculture, Sacramento, CA 95814; 5 Milk Producers Council, Ontario, CA 91761; 6 Michigan Department of Agriculture and Rural Development, Lansing, MI 48933

## Abstract

Highly pathogenic avian influenza H5N1 has infected US dairy cattle and spread throughout 19 different states since March 2024. Although within-farm transmission is thought to occur primarily through direct contact, local between-farm spread remains unknown, and is considered multifactorial and likely driven by routine business activities involving shared personnel, vehicles, and other conveyances. This investigation evaluated the presence of influenza A virus RNA in a convenience sample of milk transport trucks and other on-farm service vehicles in H5N1-affected areas in 3 states between May 2024 and April 2025. A total of 298 samples were collected from 52 vehicles, of which 38 were milk trucks and tankers from 2 dairy cooperatives. Influenza A virus RNA was detected in 1.3% (4/298) of samples: 2 from tires on a livestock hauler and a deadstock trailer in Michigan, 1 from a milk cabinet sample from a milk tanker in Michigan, and 1 from a tanker milk sample in California. Although a very small percentage of samples were positive, this investigation shows that H5N1 virus can be detected on vehicles servicing US dairy herds, highlighting a potential pathway for between-farm transmission.

## Highlights

• 298 samples from 52 vehicles were collected and tested for influenza A RNA.
• Influenza A RNA was detected in 1.3% of samples and on 7.7% of vehicles.
• Vehicles can carry detectable influenza A virus RNA.
• The findings support enhanced sanitation of vehicles visiting multiple farms.

## Graphical Abstract

**Figure:**
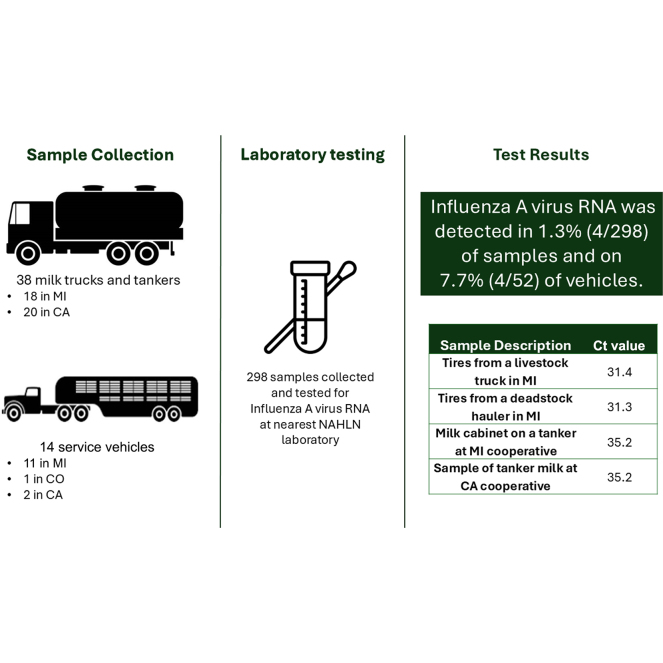
**Summary:**This investigation evaluated the presence of influenza A virus RNA on dairy transport vehicles during the 2024 to 2025 H5N1 outbreak. A total of 298 samples were collected from 52 vehicles, including milk trucks, tankers, and livestock service vehicles operating in Michigan (MI), Colorado (CO), and California (CA). Samples were tested using real-time reverse-transcription polymerase chain reaction (RT-PCR). Overall, influenza A RNA was identified in 1.3% (4/298) of samples and on 7.7% (4/52) of vehicles. Of the 4 samples with RT-PCR detections, 2 were from tires on a livestock hauler and a deadstock trailer in MI. The other 2 RT-PCR detections were from a milk cabinet sample from a milk tanker in MI and a tanker milk sample from CA. Although detections were infrequent and viral loads were low, the results demonstrate that vehicles can carry detectable influenza A virus RNA. These findings highlight the importance of sanitation practices for dairy transport. Ct = cycle threshold; NAHLN = National Animal Health Laboratory Network.

In February 2024, dairy herds in Texas and Kansas reported a nonspecific illness in lactating cows, characterized by fever, lethargy, dehydration, and an abrupt decrease in milk production. Veterinary investigations later identified H5N1 subtype influenza A virus (**IAV**) as the cause of the disease (Burrough et al., 2024). The initial infection likely resulted from spillover of H5N1 shed by infected wild birds, with subsequent cow-to-cow transmission sustaining spread within and between herds. Since its emergence, H5N1 genotypes B3.13 and D1.1 have been detected in dairy cattle across 19 US states as of December 2025 (USDA-APHIS 2025a,b). Within-herd transmission is thought to occur primarily through direct contact, and the potential for between-farm spread remains a critical concern. After the virus was introduced into Michigan through interstate animal movement, it disseminated quickly among dairy herds and subsequently into nearby poultry operations. Ongoing transmission was considered multifactorial and likely driven by routine business activities involving shared personnel, vehicles, and other conveyances (Schwabenlander et al., 2024). Among these, milk transport trucks represent a particularly plausible fomite risk, due to their daily movement between multiple dairies. Milk from infected cows has been shown to have a large amount of virus, making milk trucks and tankers an even greater concern when moving from farm-to-farm. However, empirical data on viral contamination of these vehicles remains limited. The objective of this investigation was to evaluate contamination and the potential movement of IAV via milk trucks and tankers and other farm-related vehicles.

Sampling of dairy transport vehicles for H5N1 was conducted across 3 states between May 2024 and April 2025 at various stages of each state's outbreak (Figure 1). Convenience samples of milk trucks and tankers and other vehicles were evaluated for viral contamination during an ongoing outbreak and depended on voluntary participation from producers and industry. Consequently, differences in sampling frequency, sample types, and timing occurred across states during the outbreak.

**Figure 1:**
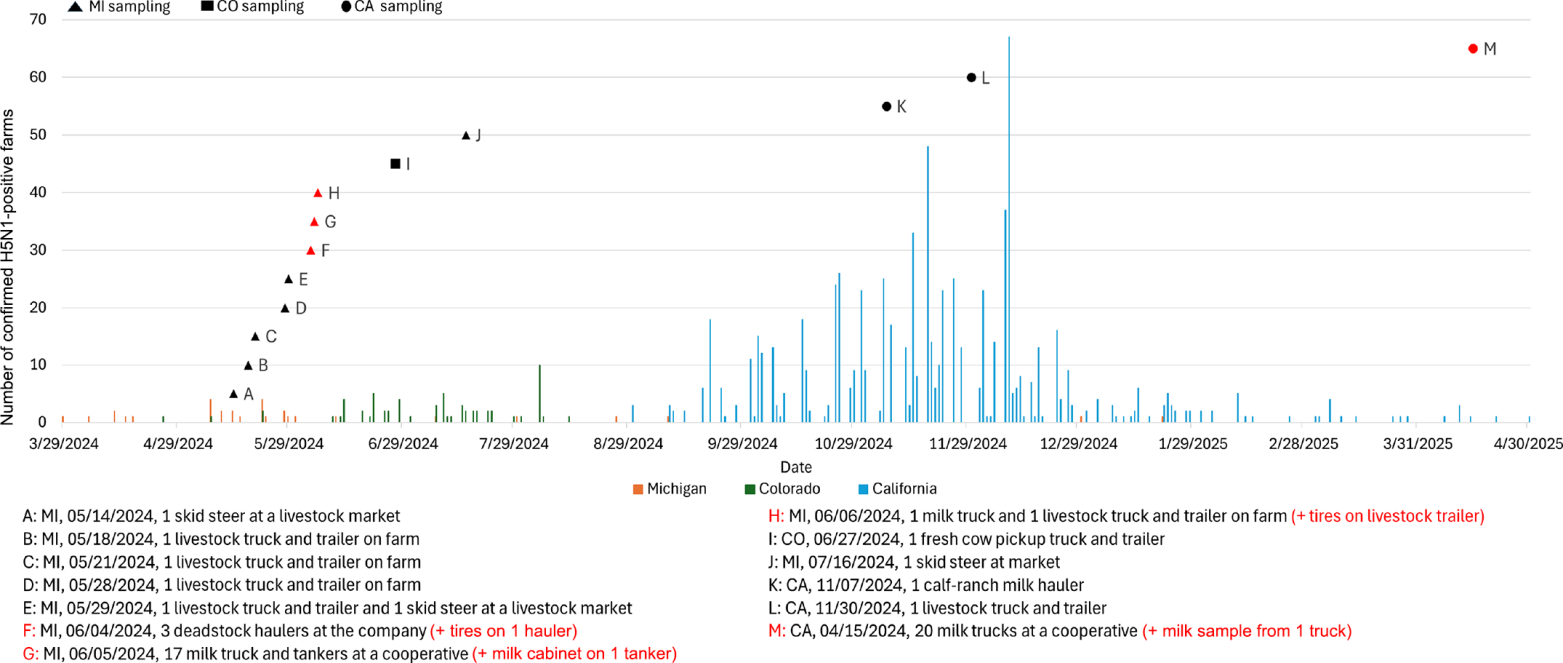
Number of newly confirmed H5N1-positive farms in Michigan (MI), Colorado (CO), and California (CA) from March 29, 2024, to August 1, 2025, with vehicle sampling dates; RT-PCR influenza A virus–positive samples are denoted in red.

In Michigan, H5N1 was first confirmed on March 29, 2024 (USDA-APHIS, 2025a) and rapidly spread to multiple dairy farms and nearby poultry premises by early April. Vehicle sampling began in mid-to-late May and included 3 deadstock haulers servicing affected dairies with sampling occurring at the company's parking lot on June 4, 2024. All haulers had completed their farm routes within the last 2 d, gone to the landfill, and returned to the company. Two of the deadstock haulers had been power-washed, following company protocol, before sample collection. Four livestock trailers with associated trucks on H5N1-affected farms were sampled between May 18, 2024, and June 6, 2024, 14 to 32 d after reported onset of clinical signs on the farms. A livestock hauler and 3 skid steers at 3 livestock markets within the affected area were sampled between May 14 and July 16, 2024. Additionally, in early June, after the peak of the outbreak, 18 milk trucks and tankers operating in the HPAI-affected region were sampled, including 1 on-farm and 17 at a local cooperative. When sampling milk transport vehicles, drivers were asked the number of stops they had made since washing the truck and whether they changed footwear between stops.

In Colorado, where the first affected dairy was reported on April 25, 2024 (USDA-APHIS, 2025a), a fresh-cow pickup truck and trailer were sampled on June 27, 2024, on a farm that reported initial clinical signs on June 17, 2024.

In California, H5N1 infections in dairy herds were first confirmed on August 30, 2024 (USDA-APHIS, 2025a). A calf-ranch milk hauler and a truck and trailer were sampled on November 7, 2024, and November 30, 2024, shortly after clinical signs were reported by producers on each farm on November 1, 2024, and November 29, 2024, respectively. Twenty milk trucks and tankers servicing affected areas were later sampled on March 26, 2025, which also occurred after the peak of the California outbreak. Milk truck drivers were asked the number of stops they had made since washing the truck and whether they changed footwear between stops.

Sampling sites on the vehicle were identified based on high-contact areas of humans, animals, and milk. Specifically, for milk trucks and tankers, samples were collected from the truck cab and tires, milk dipper or cooler, milk cabinet on the milk tanker, including hoses and valves, exterior sides of the tanker, spill-dam overflow, and tanker tires. Samples were collected from both direct-load and farm-load vehicles. In Michigan, milk truck drivers voluntarily walked through 1 of 2 footbaths filled with brain heart infusion (**BHI**) broth, which was submitted for testing. In California, a milk sample from 17 of the 18 milk tankers was also collected in addition to the truck and tanker sample locations noted. Driver footbath samples were not collected in California. For the miscellaneous farm vehicles, samples were collected from both the trucks and the trailers, and included the tires, handles and latches, floors, and walls.

Samples were collected using 3 × 3-inch (7.62 cm^2^) gauze pads. Each swab or gauze was moistened with viral transport medium (BHI) before sampling. The selected area was rubbed multiple times in a back-and-forth motion to ensure thorough contact with the surface and to maximize potential recovery. Following collection, the used gauze pads were placed into resealable plastic bags with BHI, and the swabs and gauze were soaked and the liquid fraction (normally ∼1–3 mL) removed. Samples were stored on ice and either delivered on the day of collection or shipped overnight on ice to the nearest National Animal Health Laboratory Network (**NAHLN**) laboratory.

Michigan samples were tested at the Michigan State University Veterinary Diagnostic Laboratory (Lansing, MI), and the milk truck and tanker samples were all tested at USDA's National Veterinary Services Laboratories (Ames, IA). Samples collected in Colorado were tested at Colorado State University Veterinary Diagnostic Laboratory (Fort Collins, CO), and samples collected in California were tested at the California Animal Health and Food Safety Laboratory System at the University of California–Davis (Davis, CA). Samples were refrigerated until processing. Nucleic acids were extracted following standard NAHLN protocols, and samples were tested for IAV RNA using real-time reverse-transcription PCR (**RT-PCR**). Appropriate positive and negative controls were included in each run. Results were recorded as detected or not detected for IAV RNA, with a corresponding cycle threshold (**Ct**) value for detected samples, approximating the quantity of viral particles in the sample, regardless of viability.

A total of 298 samples were collected from 52 vehicles, of which 38 were milk trucks and tankers. Influenza A virus RNA was detected in 1.3% (4/298) of samples and on 7.7% (4/52) of vehicles (Table 1). Of the 4 samples with positive RT-PCR detections, 2 were from tires on a livestock hauler and a deadstock trailer in Michigan that had been power-washed before sampling. The other 2 RT-PCR–positive samples were from a milk cabinet sample from a milk tanker in Michigan and a tanker milk sample from California (Table 2). All positive samples had a Ct value greater than 30. Only the positive milk tanker from Michigan was submitted for virus isolation. Additionally, 29 samples from the Michigan milk truck and tanker sampling had sufficient inhibition on RT-PCR, meaning that contaminating substances were present at high enough concentrations in the sample that they reduced or delayed the amplification of target nucleic acids. Therefore, virus isolation was attempted as a secondary testing method on these samples and the single positive RT-PCR sample from the Michigan milk truck testing, but no virus was recovered.

**Table 1:** Number of samples tested and number of samples RT-PCR positive for influenza A virus by sample category^1^

Sample category	Number tested by state			Total number tested	Number RT-PCR positive
	CA	CO	MI		
Tires	42	0	23	65	2
Tanker milk sample	19	0	26	45	1
Side of milk tanker	21	0	19	40	0
Milk cabinet on milk tanker	20	0	19	39	1
Outside of milk truck cab	21	0	18	39	0
Milk truck dipper	0	0	18	18	0
Milk tanker spill-dam overflow	1	0	10	11	0
Livestock trailer	0	1	12	13	0
Inside truck cab	0	0	5	5	0
Skid steer	0	0	5	5	0
PPE	0	0	5	5	0
Other	1	1	11	13	0
Total	125	2	171	298	4

1 CA = California; CO = Colorado; MI = Michigan; PPE = personal protective equipment.

**Table 2:** Sample description, location, and Ct value of 4 RT-PCR influenza A virus–positive truck samples

Sample description	Sample date	Sample location^1^	Additional information	Ct value
Truck tires from a livestock hauler	Jun. 5, 2024	MI farm	Sample collected 32 d after clinical signs reported on farm	31.4
Tires from a deadstock hauler	Jun. 4, 2024	MI deadstock truck company parking lot	Had completed a route the day of sampling and was power-washed before sampling	31.3
Milk cabinet on a milk truck	Jun. 5, 2024	MI cooperative	Direct-load truck, single farm	35.2
Sample of tanker milk	Apr. 15, 2025	CA cooperative	Had made 2 stops since the truck was washed	35.2

1 MI = Michigan; CA = California.

With respect to milk truck and tanker washing practices, drivers reported that an average of 4.8 farm stops occurred between vehicle washes, with a range of 1 to 35 stops between washes. No milk truck drivers changed their shoes between stops, but 1 driver in Michigan had bleached their tires and boots at the farm, and 1 driver had sprayed their truck and tanker tires with a disinfectant. Given that only a single positive sample was detected, no conclusions or associations could be drawn between cleaning practices and IAV RT-PCR positivity.

This investigation showed that IAV can be detected on vehicles servicing US dairy herds, highlighting a potential pathway for between-farm transmission. Additionally, the deadstock hauler with a RT-PCR–positive tire sample demonstrates that IAV can be detected on vehicles after driving on roads, which is an important factor for assessing true transmission risk. However, it is important to note that these detections may reflect environmental contamination, including exposure from wild birds or other agricultural activities, rather than direct contamination originating from the farm.

Although only a very small proportion of samples were RNA-positive, contamination was detected on multiple vehicle types, including livestock trailers, deadstock haulers, and milk tankers, indicating that a variety of routine dairy-service vehicles were contaminated with viral material. These vehicles were likely exposed to H5N1 through contaminated milk, sick cows, or carcasses on affected operations. However, it is unknown whether these vehicles were contaminated with infectious virus, as viral isolation was not completed, and all RT-PCR–positive samples exhibited high Ct values (>30), suggesting low viral genomic load. Nonetheless, even low levels of contamination on vehicles that frequent multiple operations may represent a mechanical transmission risk. Similar results have been documented in the swine and poultry industry, where service vehicles have been identified to be contaminated with pathogens such as porcine epidemic diarrhea virus, porcine reproductive and respiratory syndrome, and various avian influenza strains (Thakur et al., 2017; Yoo et al., 2021; Huneau-Salaün et al., 2022; Parker et al., 2025). The detection of IAV RNA on dairy-associated vehicles aligns with these findings and highlights the importance of biosecurity across industries.

Several limitations must be considered when interpreting these results. Samples were collected after the peak of the outbreak in each region, when viral genomic loads on farms were likely lower. In Michigan, milk samples were not collected from each milk tanker, leaving uncertainty as to whether the absence of detections reflected a true lack of contamination of the exterior of the trucks or simply a lack of virus in the milk.

Additionally, a high number of Michigan milk truck and tanker samples exhibited sufficient RT-PCR inhibition likely due to high levels of environmental contamination. Sampling from tires, metal surfaces, and tanker exteriors may be prone to RT-PCR inhibition because these vehicle surfaces can accumulate inhibitory contaminants, such as metals, environmental debris, and humic substances, that interfere with amplification (Schrader et al., 2012). Although virus isolation was attempted on these inhibited samples as well as the single RT-PCR–positive sample, no viable virus was recovered, further constraining assessment of infectious virus presence on these vehicles. Although RT-PCR for IAV is highly sensitive and specific for detecting viral RNA in other species (Spackman, 2020), inhibited samples may still yield false-negative results (Schrader et al., 2012). Therefore, these findings may underestimate viral contamination of the sampled vehicles.

Several other limitations should be considered when interpreting these findings, including the timing of sampling relative to the outbreak, limited sample size, geographic scope, and variation in sampling techniques across states. In California, only 1 milk sample tested positive, indicating that overall viral exposure from milk trucks and tankers at that time was likely minimal. Milk truck sampling was conducted only in California and Michigan and was limited to a single milk cooperative in each state, further constraining sample size and representativeness. Sampling techniques differed across states, including variation in collection methods, swab types, and handling procedures, which may have contributed to differences in sample quality. Sample dilution during collection or processing may also have reduced viral RNA concentrations below detectable limits. In addition, the interval between sample collection and processing varied, and the milk-based nature of many of these samples introduces challenges related to high levels of calcium, fat, and endogenous RNases that can compromise RNA integrity during transport and storage (Snoeck et al., 2024). Finally, virus isolation was not attempted for 3 of the 4 RT-PCR–positive samples due to high Ct values. Therefore, the infectivity of the detected viral RNA could not be assessed.

Despite these constraints, the investigation provides evidence that vehicles can carry detectable IAV RNA. These findings underscore the importance of enhanced sanitation protocols for both external and internal vehicle surfaces, including thorough cleaning with hot water, detergent, and disinfectants, applied according to manufacturer instructions, particularly for vehicles visiting multiple farms in a single day. Route management should also be taken into consideration, such as prioritizing HPAI-affected farms last on milk hauling routes, if plausible, to further mitigate risk. Increased communication between producers, haulers, and veterinarians is also critical to ensure consistent and proper adoption of biosecurity practices.

Future research should focus on determining viral viability on vehicle surfaces, characterizing persistence under different environmental conditions, and evaluating the effectiveness of cleaning and disinfection procedures. Expanded sampling across regions and vehicle types would also help refine risk estimates. Together, these efforts will inform evidence-based recommendations to reduce the potential for transmission of H5N1 virus between farms. Additionally, these findings may inform preparedness and response efforts for other emerging infectious diseases across the US dairy industry.
